# Machine learning predicting acute pain and opioid dose in radiation treated oropharyngeal cancer patients

**DOI:** 10.3389/fpain.2025.1567632

**Published:** 2025-04-04

**Authors:** Vivian Salama, Laia Humbert-Vidan, Brandon Godinich, Kareem A. Wahid, Dina M. ElHabashy, Mohamed A. Naser, Renjie He, Abdallah S. R. Mohamed, Ariana J. Sahli, Katherine A. Hutcheson, Gary Brandon Gunn, David I. Rosenthal, Clifton D. Fuller, Amy C. Moreno

**Affiliations:** ^1^ Department of Radiation Oncology, The University of Texas MD Anderson Cancer Center, Houston, TX, United States; ^2^ Department of Medical Oncology and Radiation Oncology, West Virginia University Cancer Institute, Morgantown, WV, United States; ^3^ Department of Medical Education, Paul L. Foster School of Medicine, Texas Tech Health Sciences Center, El Paso, TX, United States; ^4^ Department of Imaging Physics, The University of Texas MD Anderson Cancer Center, Houston, TX, United States; ^5^ Department of Head and Neck Surgery, The University of Texas, MD Anderson Cancer Center, Houston, TX, United States

**Keywords:** acute pain, opioid dose, radiation therapy, head and neck cancers, oral cavity and oropharyngeal cancers, machine learning, artificial intelligence

## Abstract

**Introduction:**

Acute pain is common among oral cavity/oropharyngeal cancer (OCC/OPC) patients undergoing radiation therapy (RT). This study aimed to predict acute pain severity and opioid doses during RT using machine learning (ML), facilitating risk-stratification models for clinical trials.

**Methods:**

A retrospective study examined 900 OCC/OPC patients treated with RT during 2017–2023. Pain intensity was assessed using NRS (0-none, 10-worst) and total opioid doses were calculated using morphine equivalent daily dose (MEDD) conversion factors. Analgesics efficacy was assessed using combined pain intensity and total MEDD. ML predictive models were developed and validated, including Logistic Regression (LR), Support Vector Machine (SVM), Random Forest (RF), and Gradient Boosting Machine (GBM). Model performance was evaluated using discrimination and calibration metrics, while feature importance was investigated using bootstrapping.

**Results:**

For predicting pain intensity, the GBM demonstrated superior discrimination performance (AUROC 0.71, recall 0.39, and *F*1 score 0.48). For predicting the total MEDD, LR model outperformed other models (AUROC 0.67). For predicting analgesics efficacy, the SVM achieved the highest specificity (0.97), while the RF and GBM models achieved the highest AUROC (0.68). RF model emerged as the best calibrated model with an ECE of 0.02 and 0.05 for pain intensity and MEDD prediction, respectively. Baseline pain scores and vital signs demonstrated the most contributing features.

**Conclusion:**

ML models showed promise in predicting end-of-treatment pain intensity, opioid requirements and analgesics efficacy in OCC/OPC patients. Baseline pain score and vital signs are crucial predictors. Their implementation in clinical practice could facilitate early risk stratification and personalized pain management.

## Introduction

Acute pain is one of the most common debilitating symptoms that develops during Radiation Therapy (RT), in oral cavity and oropharyngeal (OCC/OPC) cancers (1–7). Despite advancements in RT techniques improving patient outcomes, various acute adverse symptoms persist, impacting patients' quality of life (QoL) (2). These adverse symptoms have a negative impact on the patients' quality of life (QoL) (3, 8, 9). Nearly one-third of head and neck cancer (HNC) patients experience severe, uncontrolled pain (1, 10, 11). Over 90% of OCC/OPC patients report acute mouth/throat pain, with up to 80% needing opioid prescriptions for pain management (12, 13).

Managing pain in head and neck cancer (HNC) patients undergoing RT is challenging despite the World Health Organization's (WHO) analgesic ladder guidelines. The complexity of pain, its multifactorial etiology, and varying individual responses to treatment contribute to this challenge (2, 14). Opioids are commonly prescribed during RT for HNC (1, 13, 15), but their escalated doses heighten morbidity and raise concerns about side effects and substance abuse (16). These challenges in pain management not only complicate care but also have a detrimental impact on the QoL for the survivors within this cancer population. Approximately 45% of long-term HNC survivors report chronic pain, with more than 10% exhibiting severe chronic pain with chronic opioid usage (17). The long-term opioid usage raises the risks of opioid dependence and drug addiction which may lead to patient death (18–20). Overuse of opioids during RT exacerbates patient care complexity and risks overall health outcomes (21).

Artificial Intelligence and machine learning (AI/ML) models are being studied for risk stratification in pain medicine and opioid use (22). These models aim to optimize pain management and assist in personalized treatments through risk stratification and decision-making (22). For example, Chao et al. used ML algorithms to identify chest wall pain induced by RT in non-small cell lung cancer (NSCLC) patients treated with Stereotactic Body Radiation Therapy (23) and Olling et al. generated ML predictive models for predicting pain while swallowing (odynophagia) during RT in NSCLC (24). While approximately 44 studies have explored ML models to predict cancer pain, no studies have investigated the role of ML models in pain prediction in HNC and how they can aid in guiding decisions related to the use of opioids in these individuals (25).

The primary objective of the present study is to address this gap in knowledge by (a) comparing the performance of various ML algorithms as predictive models for predicting acute pain levels, (b) projecting opioid doses at the end of RT in OCC/OPC patients and (c) identifying the importance of relevant clinical predictors in classifying acute pain and predicting the required opioid dosages.

## Materials and methods

### Patient data

**Figure 1:**
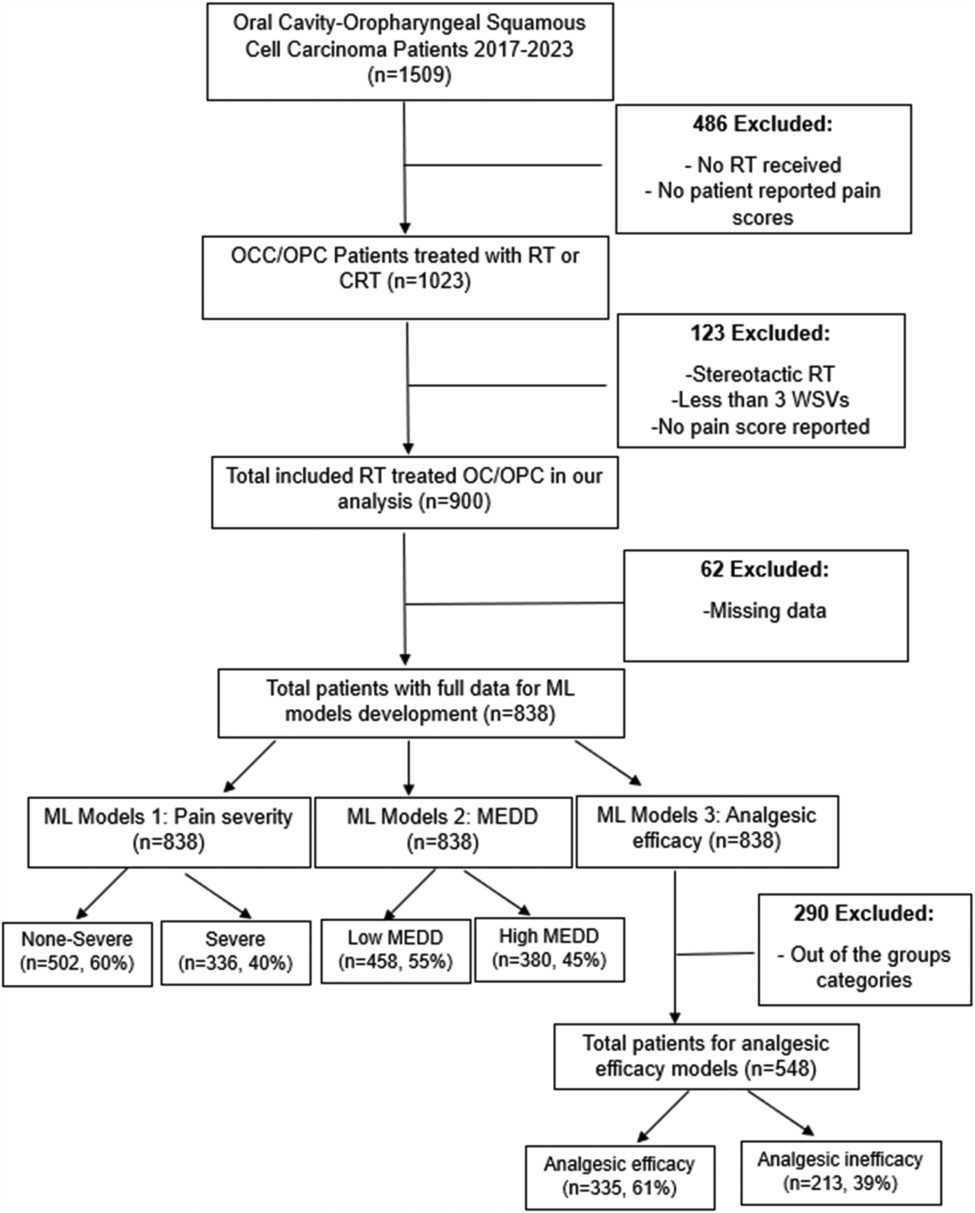
Flowchart of study population.

A retrospective study was conducted using a cohort of oral cavity cancer, oropharyngeal cancer and unknown primary cancer patients treated with RT at our institution from 2017 to 2023. Since most unknown primary cancers end up being oropharyngeal cancer (OPC) or oral cavity cancer (OCC), they were included in our study. The study has been approved by The University of Texas MD Anderson Cancer Center (MDACC) Institutional Review Board (IRB) (2024-0002). Patients selected for this study met the following inclusion criteria: (1) age ≥18 years, (2) a pathologic diagnosis of squamous cell carcinoma (SCC) of the oral cavity, oropharynx, or unknown primary, (3) treatment with RT or chemoradiation therapy (CRT) for curative intent, and (4) RT modalities included photons [intensity-modulated radiotherapy (IMRT) and volumetric modulated arc therapy (VMAT)] and proton therapy (IMPT). Patients were excluded if had any of the following criteria: (1) no patient reported pain scores available, (2) received stereotactic RT, (3) had fewer than three weekly see visits (WSVs) during RT, or (4) did not report pain scores at the end of RT. In the development of the ML models, patients with any missing data were excluded. Flow chart of the study design is illustrated in Figure 1.

### Predictors

Clinical data extracted from the electronic health record system included patient demographics, social history (smoking, alcohol, drug abuse), tumor and staging characteristics, cancer therapy details (systemic therapy, surgery, RT), vital signs (weight and heart rate), medications, and baseline and last on-treatment visit (i.e., weekly see visit, WSV) acute pain scores. Delta changes in weight were calculated as follows: [(last WSV weight—baseline weight)/baseline weight]*100.

### Outcomes

Our ML models aimed to predict three endpoints: end-of-RT pain intensity, opioid usage and status of analgesic efficacy at the end of RT.

Pain intensity, rated on a scale of 0–10 during nursing visits, was categorized into non-severe (0–6) and severe (7–10) based on established literature (2, 26, 27) and clinical thresholds where a score of 7 or higher indicates heightened risk for uncontrolled pain (28, 29).

Opioid usage was measured as the total morphine equivalent daily dose (MEDD). Total MEDD at the last visit was determined according to the Centers for Disease Control and Prevention (CDC) guidelines (30–33). The total MEDD was calculated as follows: the unit dose of all opioids (i.e., tramadol, hydrocodone, oxycodone, morphine, methadone, transdermal fentanyl) prescribed during the last WSV were collected and multiplied by the prescribed frequency and their CDC-based MEDD conversion factors [hydrocodone = 1, hydromorphone = 4, morphine = 1, oxycodone = 1.5, tramadol = 0.1, transdermal fentanyl = 2.4 and methadone according to the dose (1–20 mg/day = 4, 21–40 mg/day = 8, 41–60 mg/day = 10 and ≥61–80 mg/day = 12)] (30–32). For analysis, MEDD was dichotomized into low (<50 mg/day) vs. high (≥50 mg/day) categories, with 50 mg/day chosen based on our cohort's mean MEDD and CDC guidance (30, 33).

Analgesic efficacy at the end of RT was dichotomized into two classes: analgesic efficacy (non-severe pain and low MEDD) and analgesic inefficacy (severe pain and high MEDD). Identifying analgesic efficacy preemptively in HNC patients undergoing RT can facilitate better patient management and outcomes (18, 19, 29, 34, 35).

### Descriptive statistics

Differences in patient characteristics between pain classes and total MEDD were compared using the Chi-square test for categorical variables and Wilcoxon test for numeric variables. A 2-sided *P*-value less than 0.05 was considered statistically significant.

### Classification models

The full dataset was randomly split with stratification, into training dataset (70%) and test dataset (30%). Categorical variables elements were converted into numerical values. Patients with any missing variables and variables with a percentage of missing data more than 10% were excluded. Normalization of numeric variables, done for the training and test datasets separately, was performed using the robust scaler method (36).

#### Model training

Four classification models were trained in Python using ML algorithms including Logistic Regression (LR), Support Vector Machine (SVM), Random Forest (RF), and Gradient Boosting Machine (GBM). All models were initialized with default settings with hyper-parameter optimization performed on the LR, RF and GBM models following a manual grid search approach. The pain intensity prediction models were trained as follows: the LR model was trained with the sklearn.linear_model.LogisticRegression function (penalty = “l2”, C = 1.0, solver = “lbfgs”, max_iter = 100, fit_intercept = True, random_state = None). the RF model was trained with the sklearn.ensemble.RandomForestClassifier function (n_estimators = 100, random_state = 10) and the GBM model was trained with the sklearn.ensemble.GradientBoostingClassifier (n_estimators = 100, learning_rate = 0.1, max_depth = 2, random_state = 12) function. For the MEDD prediction models, the same hyperparameters were used for LR and SVM prediction models. The RF (n_estimators = 100, random_state = 10, max_depth = 3, min_samples_leaf = 3) and GB (n_estimators = 100, learning_rate = 0.1, max_depth = 2, random_state = 12, min_samples_split = 3) models were trained with slightly different hyperparameters to ones in the corresponding pain intensity models.

#### Model evaluation

Model validation was conducted using a ten-fold cross-validation (CV) approach. Model performance was assessed on the test dataset in terms of discrimination performance and model calibration. The discriminative ability was measured using the following metrics: area under the receiver operating curve (AUROC), recall, precision, and *F*1 score. Statistical significance of the observed differences in AUROC scores between models was assessed with the DeLong test using the R pROc package (37, 38). Calibration performance was assessed with the reliability curve and the Expected Calibration Error (ECE) (39, 40). ECE was calculated as the mean absolute difference between the observed and predicted probabilities across predefined bins (39, 40). A lower ECE value indicates better calibration (39).

#### Feature importance

The determination of feature importance for the highest performing model (i.e., the highest AUROC) was computed to elucidate the individual contributions of each predictor variable to the model's overall performance. For the GBM and RF models, feature importance was calculated through bootstrapped resampling and the calculation of both mean and standard deviation across 100 runs. For the LR and the SVM classifiers, evaluation of feature importance was conducted through the application of permutation feature importance analysis. The resulting feature importance values were then sorted and visualized using a boxplot, providing a comprehensive view of the distribution of feature importance.

#### Decision curve analysis (DCA)

Decision Curve Analysis was created for the highest performance models. After training the model, predicted probabilities for the test set were extracted. The net benefit at different threshold probabilities were computed after computes the true positives (TP) and false positives (FP), using the following formula (Equation 1) (41).

(1) $$\mathrm{Net}\;\mathrm{Benefit}=\frac{\mathrm{TP}}{N}\left(\frac{\mathrm{FP}}{N}\times \frac{\mathrm{threshold}}{1-\mathrm{threshold}}\right)$$

We used 50 evenly spaced thresholds from 0.01 to 0.5. Baseline comparisons used: “Treat All” assumes everyone gets treatment (Net Benefit = prevalence). “Treat None” assumes no one gets treatment (Net Benefit = 0). Decision Curve Analysis (DCA) graphs were plotted and net benefit values for different thresholds were calculated (41).

Scikit-learn packages were used for ML modeling, validation, and evaluation. All statistical analyses were performed using Python 3.12, JMP PRO 15 and R studio version 4.0.5.

## Results

### Patients characteristics

A total of 900 patients with OCC (*n* = 100, 11%), OPC (*n* = 772, 86%) or unknown primary (*n* = 28, 3%) were included in our study. Data for a total of fifteen variables were collected. Table 1 provides a summary of the cohort characteristics and the results of the Chi-square and Wilcoxon tests.

**Table 1:** Patients characteristics stratified by acute pain intensity and total MEDD.

Variable	*n*	SD/%	None-severe pain	Severe pain	*P*-value	Low MEDD	High MEDD	*P*-value
Total	900							
Age (SD)	60.65	9.8			0.0004*			0.008*
Sex (%)								
Males	766	(85%)	459 (51%)	307 (34%)	0.27	416 (46%)	350 (39%)	0.009*
Females	134	(15%)	87 (10%)	47 (5%)		89 (10%)	45 (5%)	
Race (%)								
White or Caucasian	814	(90%)	497 (55%)	317 (35%)	0.3	459 (51%)	355 (39%)	0.9
Black or African American	25	(3%)	10 (1%)	15 (2%)		15 (2%)	10 (1%)	
Asian	16	(2%)	12 (1.3%)	4 (0.7%)		8 (1%)	8 (1%)	
American Indian or Alaskan Native	4	(0.4%)	2 (0.2%)	2 (0.2%)		2 (0.2%)	2 (0.2%)	
Other/unknown	41	(4.6%)	25 (2.7%)	16 (1.3%)		21 (2.3%)	20 (2.3%)	
Smoking (%)								
Current smoker	76	(8.4%)	46 (5.1%)	30 (3.3%)	0.95	41 (4.6%)	35 (3.8%)	0.17
Former smoker	352	(39.2%)	216 (24.1%)	136 (15.1%)		185 (20.6%)	167 (18.6%)	
Never smoker	471	(52.3%)	284 (31.5%)	187 (20.8%)		278 (30.9%)	193 (21.4%)	
NA	1	(0.1)						
Alcohol (%)								
Yes	618	(69.4%)	371 (41.6%)	247 (27.7%)	0.67	354 (39.8%)	264 (29.6%)	0.175
No	273	(30.6%)	168 (18.9%)	105 (11.8%)		143 (16%)	130 (14.6%)	
NA	9	(1%)						
Drug abuse (%)								
Yes	204	(22.7%)	93 (10.3%)	111 (12.4%)	<0.0001*	97 (10.7%)	107 (12%)	0.007*
No	686	(76.2%)	445 (49.9%)	241 (27.1%)		400 (44.2%)	286 (32%)	
NA	10	(1.1%)						
Clinical-T stage (%)								
Tx	10	(1%)	10 (1%)	0 (0%)	0.111	7 (0.7%)	3 (0.3%)	0.319
T0	53	(6%)	33 (4%)	20 (2%)		34 (4%)	19 (2%)	
T1	279	(31%)	170 (19%)	109 (12%)		164 (18%)	115 (13%)	
T2	297	(33%)	176 (19.6%)	121 (13.4%)		166 (18%)	131 (15%)	
T3	147	(16%)	89 (10%)	58 (6%)		72 (8%)	75 (8%)	
T4	113	(13%)	68 (8%)	45 (5%)		61 (7%)	52 (6%)	
Clinical-N stage (%)								
NX	4	(0.4%)	2 (0.2%)	2 (0.2%)	0.63	2 (0.2%)	2 (0.2%)	0.663
N0	134	(15%)	85 (10%)	49 (5%)		83 (9%)	51 (6%)	
N1	445	(49.4%)	259 (28.8%)	189 (20.6%)		246 (27.2%)	199 (22.2%)	
N2	286	(32%)	181 (20%)	105 (12%)		158 (18%)	128 (14%)	
N3	31	(3.2%)	19 (2.1%)	12 (1.1%)		16 (1.6%)	15 (1.6%)	
Primary tumor type (%)								
Oral cavity	100	(11%)	70 (7.7%)	30 (3.3%)	0.049*	63 (7%)	37 (4%)	0.328
Oropharynx	772	(86%)	456 (51%)	35%)		427 (47%)	345 (39%)	
Unknown primary	28	(3%)	20 (2.2%)	8 (0.8%)		15 (1.6%)	13 (1.4%)	
Chemotherapy (%)								
Yes	641	(71%)	374 (41.6%)	267 (29.4%)	0.024*	355 (39%)	286 (32%)	0.481
No	259	(29%)	172 (19%)	87 (10%)		150 (17%)	109 (12%	
Surgery (%)								
Yes	283	(31%)	196 (21%)	87 (10%)	0.0003*	180 (20%)	103 (11%)	0.002*
No	617	(69%)	350 (39%)	267 (30%)		325 (36%)	292 (32%)	
Proton therapy (%)								
Yes	143	(16%)	81 (9%)	62 (7%)	0.28	74 (8%)	69 (8%)	1.3
No	757	(84%)	465 (52%)	292 (32%)		431 (48%)	326 (36%)	
Pre-RT pain (mean, SD)	2.2	2.8			<0.0001*			<0.0001*
Change in weight (mean%, SD)	−6.6	5.8			0.13			0.002*
Change in pulse (mean, SD)	13.8	17.3			0.001*			0.29
Total MEDD (mean, SD)	52	46			<0.0001*			
Last week Pain score (mean, SD)	5.3	2.7						<0.0001*

*n*, number; SD, standard deviation.

* Significant difference <0.05.

### Model performance

#### Models for predicting acute pain intensity at the end of RT in OCC/OPC

**Figure 2:**
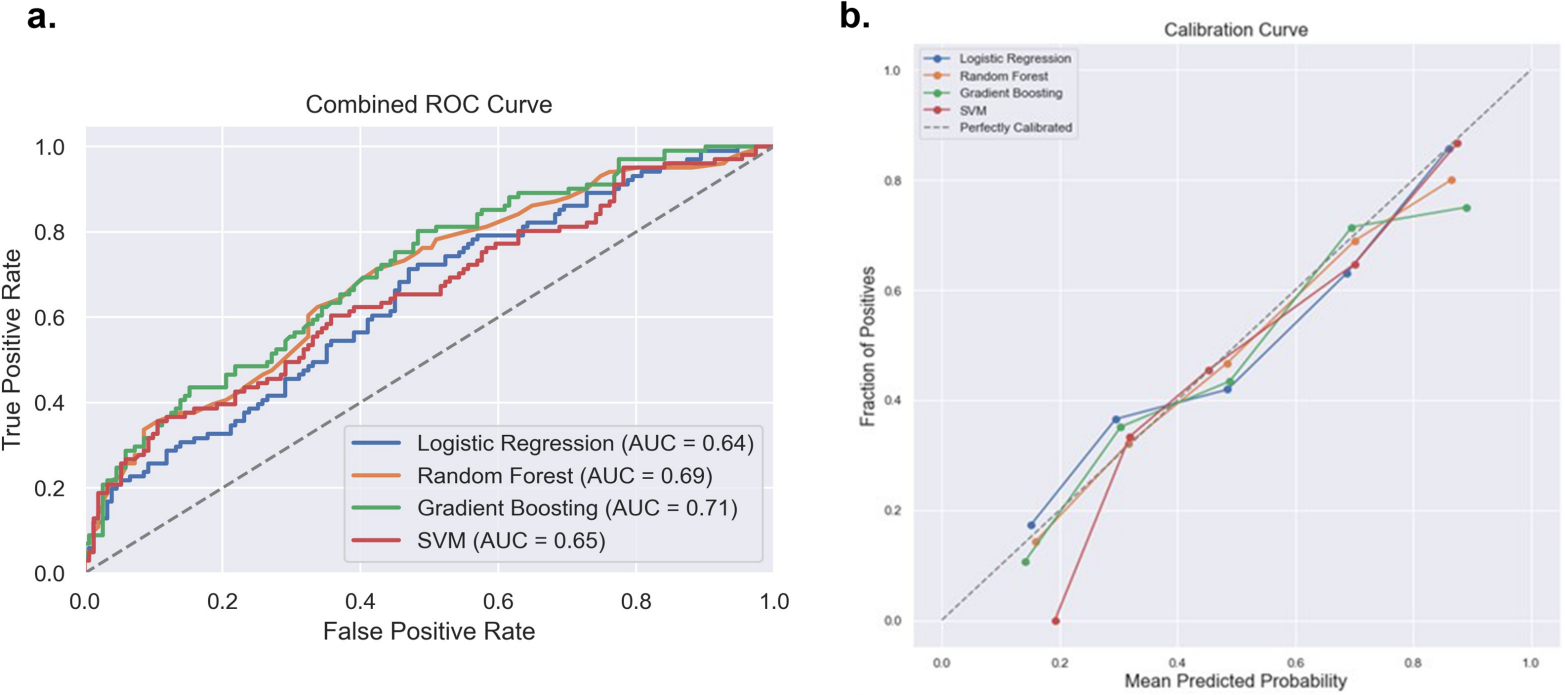
Comparison of the four prediction models [logistic regression, random forest, gradient boosting machine and support victor machine (SVM)] for acute pain intensity prediction. **(a)** Receiver operating curve area under the curve (AUROC) values for the four models on the test dataset. (**b)** Calibration curve to compare the mean predicted probability and the fraction of positives for the four models.

After exclusion of patients with missing data, data from a total of 838 patients with non-severe pain 60% (*n* = 502) and severe pain 40% (*n* = 336) was used to train the pain intensity prediction models. Table 2 summarizes the model discrimination performance results. The results of the four ML models for predicting pain intensity by the end of RT showed that GBM had the highest AUROC (0.71), while the AUROC of the RF, SVM and LR models were 0.69, 0.65 and 0.64, respectively (Figure 2a). However, no statistically significant differences were detected in AUROC scores between different models (DeLong test results are summarized in Supplementary Table S1). The SVM classifier demonstrated the highest precision (0.95) but falls behind in sensitivity (0.23) and overall *F*1 Score (0.35).

**Table 2:** Discrimination metrics and the performance of the models predicting acute pain intensity by the end of RT.

Model	AUC	Recall	Precision	*F*1 score
Logistic regression (LR)	0.6442	0.3366	0.7881	0.4072
Random forest (RF)	0.6895	0.3762	0.8411	0.4663
Gradient boosting (GB)	0.7085	0.3861	0.8609	0.4845
Support vector machine (SVM)	0.6536	0.2277	0.9470	0.3485

With regards to model calibration, the RF model showed the best performance (ECE 0.0228), followed closely by the SVM (ECE 0.0342), LR (ECE 0.0436) and GBM (ECE 0.0589). Thus, the RF model provided the most reliable, well-calibrated probability estimates compared to the other models tested. Reliability plots are shown in Figure 2b.

**Figure 3:**
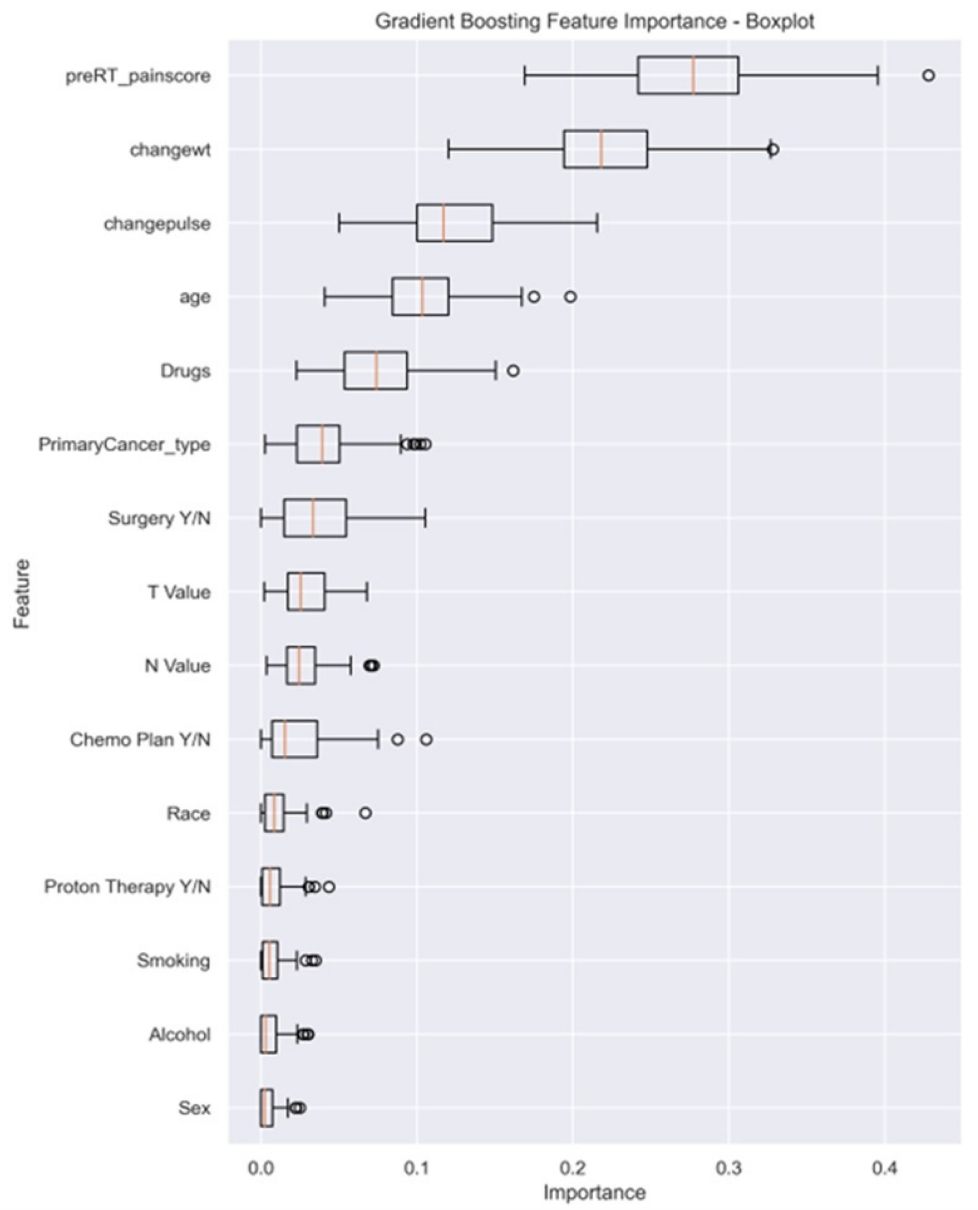
The box plot visually summarizes the distribution of feature importance obtained from a GBM. Pre-RT_painscore: pre-RT pain score; changewt: change in weight; changepulse: change in pulse (HR), Drugs (drug abuse history), primaryCancer_Type: primary cancer type (OC, OPC or unknown primary); Chemo Plan Y/N: chemotherapy plan Yes/No; T value: clinical T stage, N value: clinical N stage, Y/N: Yes or No.

Feature importance analysis from the GBM classifier, demonstrated in Figure 3, revealed key factors influencing the prediction of pain intensity by the end of RT. Baseline pre-RT pain score and changes in weight emerged as the most crucial contributors, emphasizing the significance of the initial pain levels and weight alterations in predicting acute pain by the end of RT (mean importance: 0.244 ± 0.038 and 0.214 ± 0.031, respectively). Additionally, changes in heart rate (i.e., pulse) (mean 0.147 ± 0.028), age (mean 0.123 ± 0.026), and drug abuse (mean 0.055 ± 0.018), exhibited considerable importance.

#### Models for predicting total MEDD at the end of RT in OCC/OPC

**Figure 4:**
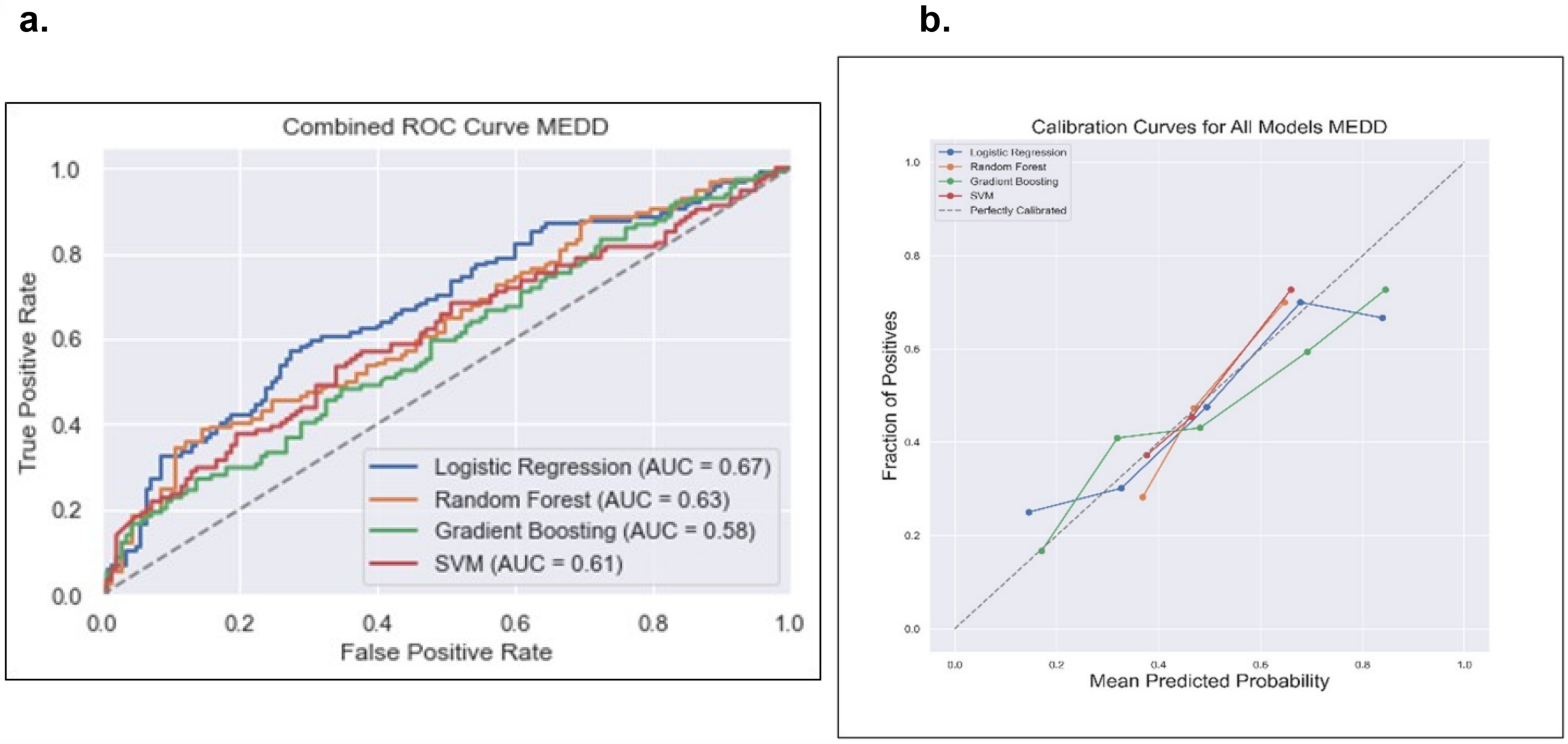
Comparison of the four prediction models [logistic regression, random forest, gradient boosting and support victor machine (SVM)] for the total MEDD prediction. **(a)** Receiver operating curve, area under the curve (AUC) values for the four models in testing dataset. **(b)** Calibration curve to compare the mean predicted probability and the fraction of positives for the four models.

Data from a total of 838 patients with low MEDD (*n* = 458, 55%) and high MEDD (*n* = 380, 45%) were used to train the MEDD prediction models. Model discrimination performance results are summarized in Table 3. The LR model outperformed the other models (AUROC 0.67), indicating its effectiveness in the discrimination performance for predicting the total MEDD at the end of RT (Figure 4a). RF showed AUC score of (0.63). GBM showed a better balance between precision (0.52) and recall (0.42) while the lowest AUC (0.58). The SVM model achieved the highest precision (0.73) but lower recall (0.19) (Table 3). Statistically differences in AUROC scores were found between the LR and GBM models (*P* = 0.007, 95% CI 0.02–0.128), SVM and GBM models (*P* = 0.02, 95% CI −0.126 to −0.011) and RF and GBM models (*P* = 0.019, 95% CI −0.09 to −0.008). DeLong test results are summarized in Supplementary Table S2.

**Table 3:** Discrimination metrics and the performance of the models predicting total MEDD by the end of RT.

Model	AUC	Recall	Precision	*F*1 score
Logistic regression	0.6693	0.5000	0.6196	0.5534
Random forest	0.6295	0.2456	0.6512	0.3567
Gradient boosting	0.5847	0.4211	0.5217	0.4660
Support vector machine	0.6066	0.1930	0.7333	0.3056

The calibration analysis of the models revealed that the RF model emerged as the top-performing model with the lowest ECE of 0.0569; the GBM model followed closely with an ECE of 0.0790 while the SVM model exhibits a higher ECE of 0.1588. These findings emphasized that RF is a particularly reliable model in providing well-calibrated probability estimates for the classification task of low MEDD vs. high MEDD. Calibration plots of the four models are illustrated in Figure 4b.

**Figure 5:**
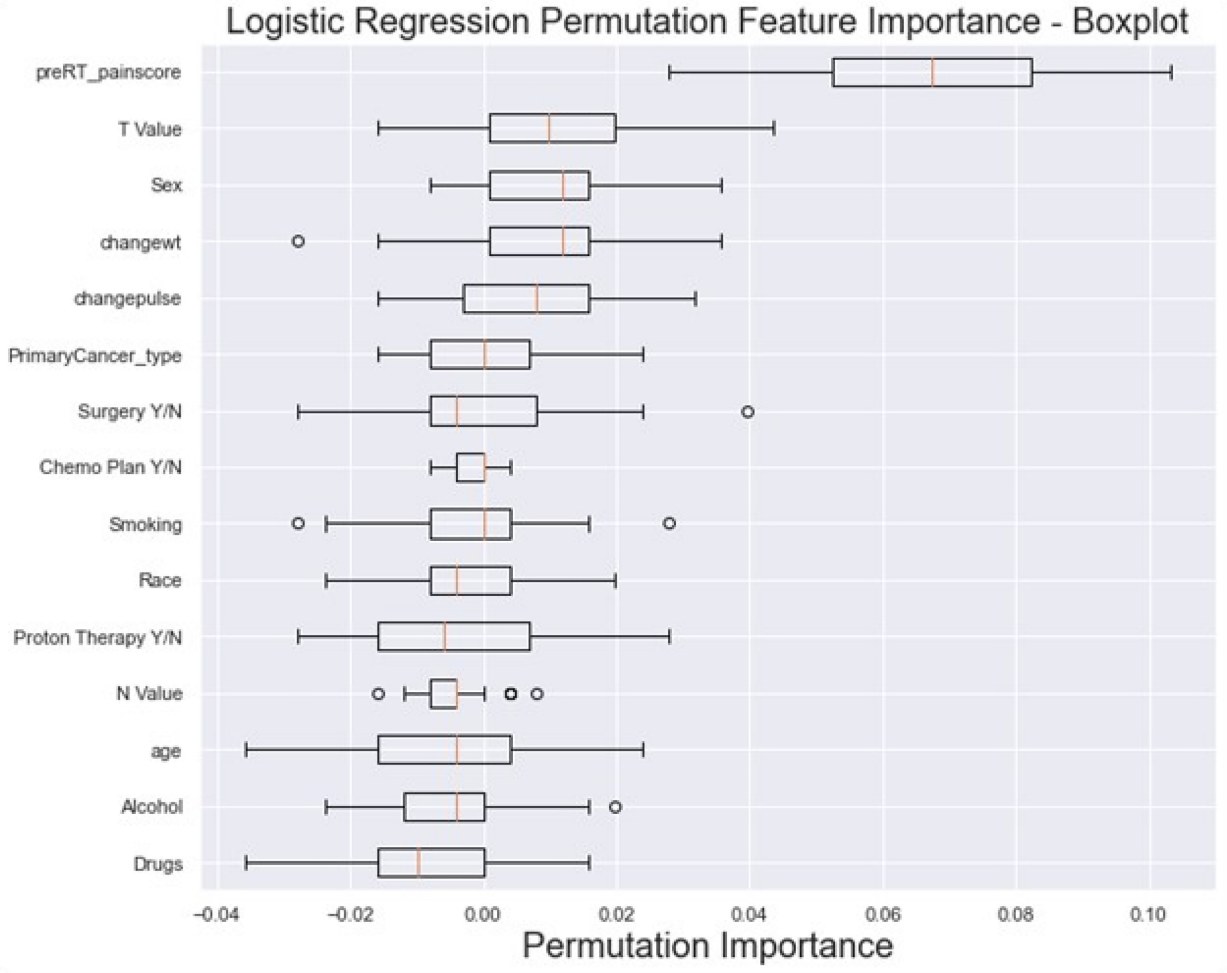
The box plot visually summarizes the distribution of LR permutation feature importance. Pre-RT_painscore: pre-RT pain score; changewt: change in weight; changepulse: change in pulse (HR), Drugs (drug abuse history), primaryCancer_Type: primary cancer type (OC, OPC or unknown primary); Chemo Plan Y/N: chemotherapy plan Yes/No; T value: clinical T stage, N value: clinical N stage, Y/N: Yes or No.

Feature importance results from the LR model permutation analysis, demonstrated in Figure 5, revealed that the most influential features include the “baseline pre-RT pain score” (mean 0.066 ± 0.021), clinical T stage (0.01 ± 0.013) and sex (0.009 ± 0.01). Conversely, features like drug abuse (−0.009 ± 0.011), alcohol (−0.005 ± 0.01) and age (−0.004 ± 0.014) had low contributions.

#### Models for predicting analgesic efficacy at the end of RT in OC/OPC

**Figure 6:**
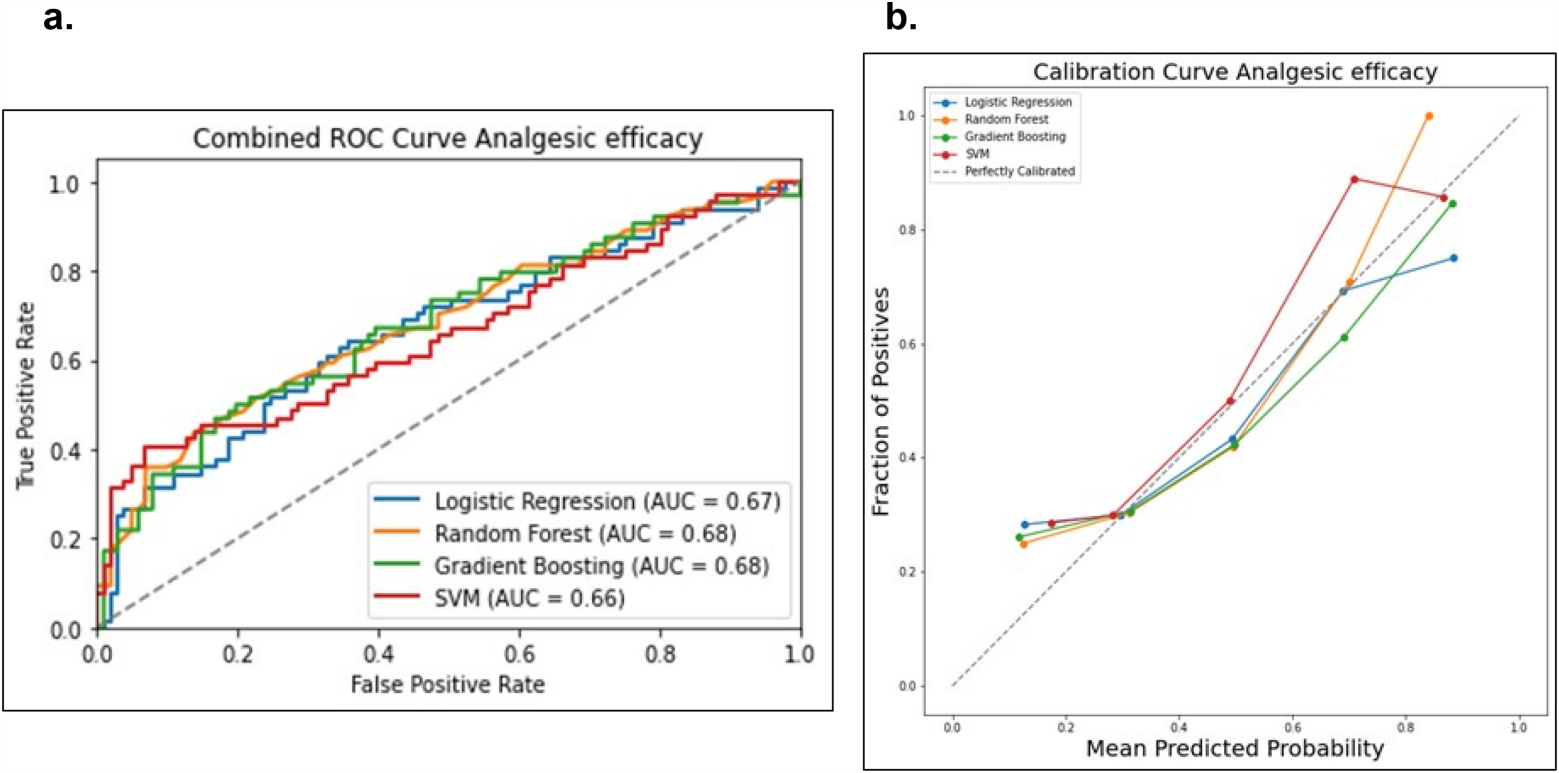
Comparison of the four prediction models (logistic regression, random forest, gradient boosting machine and support victor machine) for the analgesic efficacy status. **(a)** Receiver operating curve area under the curve (AUROC) values for the four models on the test dataset. **(b)** Calibration curve to compare the mean predicted probability and the fraction of positives for the four models.

Data from a total of 548 patients with analgesic efficacy (*n* = 335, 61%) and analgesic inefficacy (*n* = 213, 39%) were included in the analgesic efficacy prediction models. Model discrimination performance results are summarized in Table 4 and Figure 6a. All four models resulted in very similar AUROC values (RF 0.68, GBM 0.68, LR 0.67 and SVM 0.66) with no statistically significant differences (DeLong test results in Supplementary Table S3). Similarly, all four models showed a good calibration with ECE values of 0.0636 (SVM), 0.0684 (GBM), 0.0715 (LR) and 0.0756 (RF) (Figure 6b).

**Table 4:** Discrimination metrics and the performance of the models predicting analgesic efficacy by the end of RT.

Model	AUC	Sensitivity	Specificity	*F*1 score
Logistic regression	0.6658	0.3594	0.8317	0.4423
Random forest	0.6832	0.4531	0.8317	0.5273
Gradient boosting	0.6802	0.4375	0.8515	0.5234
SVM	0.6581	0.3125	0.9703	0.4598

**Figure 7:**
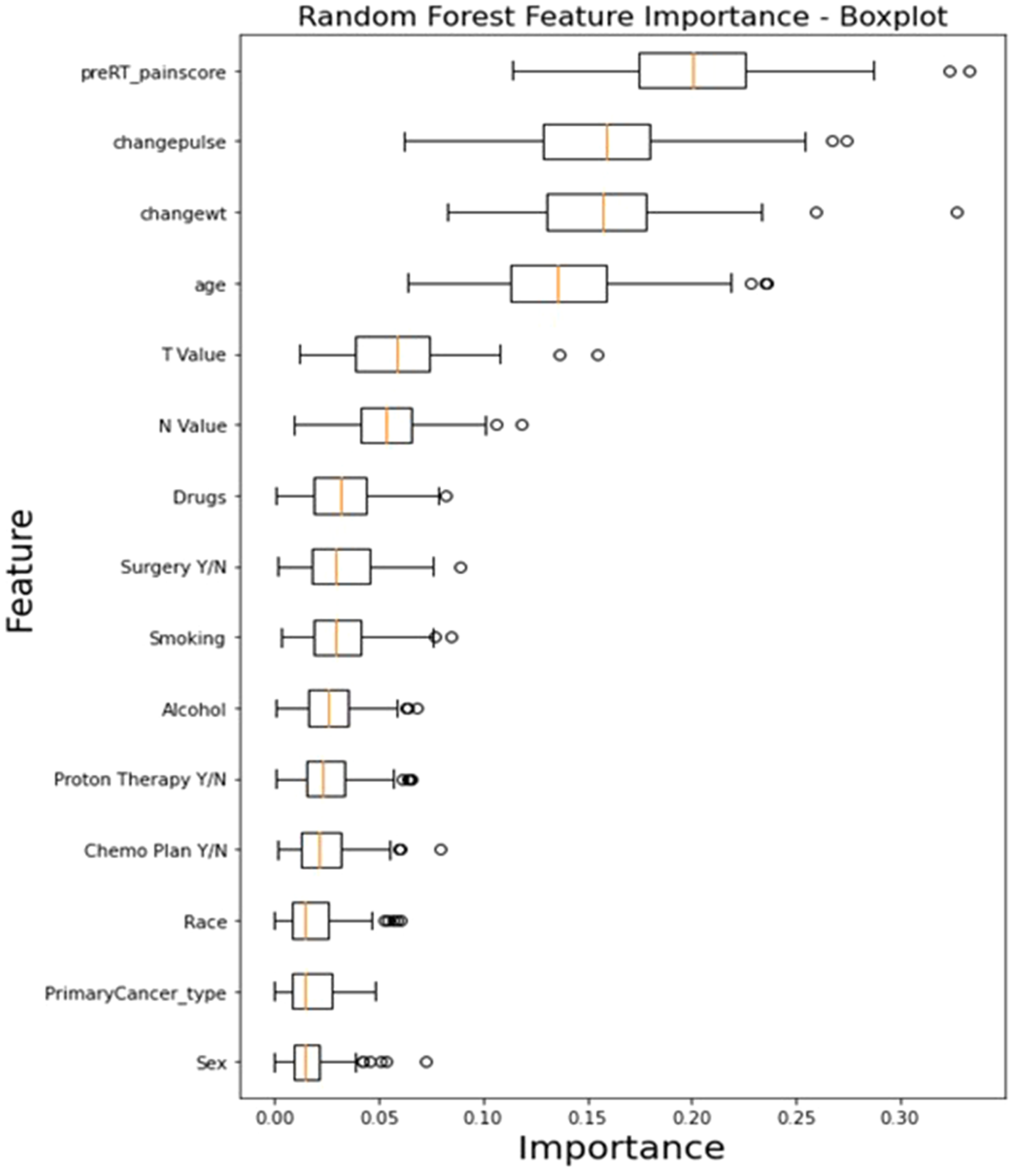
The box plot visually summarizes the distribution of RF feature importance. Pre-RT_painscore: pre-RT pain score; changewt: change in weight; changepulse: change in pulse (HR), Drugs (drug abuse history), primaryCancer_Type: primary cancer type (OC, OPC or unknown primary); Chemo Plan Y/N: chemotherapy plan Yes/No; T value: clinical T stage, N value: clinical N stage, Y/N: Yes or No.

Feature importance analysis results for the RF model, demonstrated in Figure 7, revealed that the top features that influenced the model include baseline pre-RT pain score (0.1696 ± 0.017), change in weight (0.1686 ± 0.01), and change in pulse (0.1565 ± 0.01), indicating their significant impact on the model's predictions. Other notable features include age (0.1439 ± 0.008), T stage (0.0665 ± 0.005), and N stage (0.0545 ± 0.005). On the other hand, features like the primary cancer type (0.0169 ± 0.003), sex (0.0175 ± 0.003), and race (0.0193 ± 0.003) exhibited lower importance.

### Decision curve analysis (DCA) and clinical decision optimization

**Figure 8:**
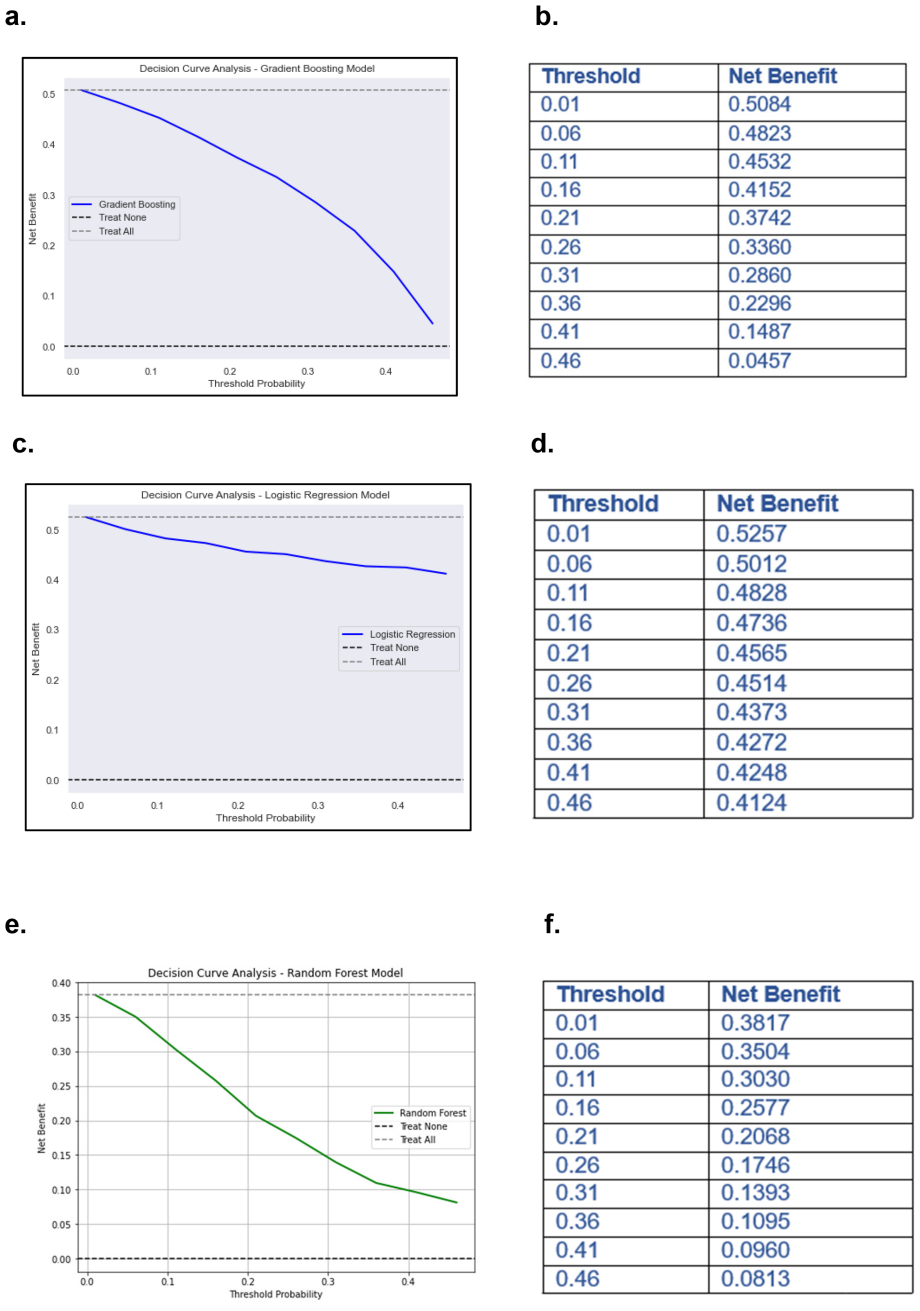
Decision curve analysis (DCA) and net benefits values of ML models. **(a)** DCA of GBM predicting pain severity. **(b)** Net benefit values at different threshold of DCA of GBM predicting pain intensity. **(c)** DCA of LR predicting MEDD. **(d)** Net benefit values at different threshold of DCA of LR predicting MEDD. **(e)** DCA of RF model predicting analgesic efficacy. **(f)** Net benefit values at different threshold of DCA of RF predicting analgesic efficacy.

For pain intensity prediction models, the DCA results for the GBM, demonstrated the model's clinical utility in identifying high-risk patients who may require proactive pain management interventions (Figure 8a). At lower threshold probabilities (e.g., 0.01–0.16), the net benefit remains relatively high (0.5084–0.4152), indicating that the model is effective in early identification of patients at risk of severe pain. This suggests that even at a low probability threshold, using the model to guide clinical decisions would result in a meaningful net benefit compared to treating all patients or treating none. As the threshold increases (e.g., 0.21–0.31), the net benefit gradually declines (0.3742–0.2860) (Figure 8b). This reflects the model's increasing specificity; it prioritizes patients with a higher predicted probability of severe pain while reducing unnecessary interventions for those at lower risk. Beyond a 0.36 threshold, the net benefit continues to decrease (0.2296–0.0457 at 0.46), suggesting that while the model still outperforms the “Treat None” strategy, its advantage over “Treat All” diminishes at higher thresholds. This could imply that at very high probabilities, fewer patients are classified as high-risk, potentially leading to under-treatment of some patients who may still develop severe pain.

For models predicting MEDD, the DCA of the LR model for predicting MEDD provided valuable insights into its clinical utility (Figure 8c). The net benefit values, calculated across different threshold probabilities, highlighted the model's performance in distinguishing between high and low MEDD predictions. At lower thresholds (e.g., 0.01), the model yields a higher net benefit, suggesting that it is more favorable for identifying patients who may require higher pain management (high MEDD). As the threshold increases, the net benefit gradually decreases (0.4124 at 0.46) (Figure 8d), indicating that the model may become less effective at predicting MEDD and may result in more false positives.

Models predicting analgesic efficacy, the DCA for the RF model illustrated how net benefits vary with different threshold probabilities when predicting analgesic efficacy for patients undergoing RT (Figure 8e). At lower thresholds (e.g., 0.01), the model shows a higher net benefit of 0.3817, suggesting that it is more effective at identifying patients who will respond well to increased pain management interventions. However, as the threshold increases, the net benefit decreases, reaching its lowest point of 0.0813 at a threshold of 0.46 (Figure 8f). This decline indicates that the model becomes less effective at accurately predicting analgesic efficacy as the threshold increases, potentially leading to false positives and unnecessary interventions.

## Discussion

Acute pain in OCC/OPC patients during RT is challenging due to radiation-induced toxicities (e.g., oral mucositis, dermatitis, dysphagia), its multifactorial nature, and the absence of data-driven tools for pain management. This exploratory study fills this gap by using ML to predict pain and opioid use during RT in this cancer population.

GBM emerged as the best model for pain intensity prediction while LR outperformed other models in predicting total MEDD and RF performed best in predicting analgesic efficacy. Although model calibration is vital for reliable clinical ML models (42, 43), few studies focused on evaluation of the calibration of the classification models investigated in the clinical settings (25, 39, 42). Our results showed good calibration of all developed models for predicting acute pain intensity, MEDD, and analgesic efficacy in OCC/OPC patients undergoing RT. These results highlight the potential of ML, especially the GBM and RF algorithms, in improving outcomes for OCC/OPC patients undergoing RT through early risk stratification and personalized pain management (25, 39, 42, 43).

Chao et al. (2028) used Decision Trees and RF models to predict chest wall pain induced by RT in NSCLC patients, showing predictive accuracy (23). Olling et al. (2018) applied LR, SVM, and Generalized Linear Models to predict swallowing pain during RT in lung cancer patients, illustrating ML effectiveness (24). Our study further supports ML efficacy in predicting acute pain, opioid dosage, and analgesic efficacy in OC/OPC patients post-RT.

Baseline pain intensity and vital signs were identified as high-risk predictors for cancer-related pain (1, 44). In a previous study, we established a correlation between vital signs, baseline pain scores, and the pain intensity during RT in OCC/OPC patients (1). Uncontrolled pain not only contributes to challenges in chewing and swallowing, leading to weight loss, but also exerts a broader impact on patients' physiological functions. Elevated pain levels are associated with increased heart rates and changes in blood pressure (1). Bendall et al., demonstrated an association between vital signs and acute pain (45) and Moscato et al. developed an automatic pain assessment tool based on physiological signals recorded by wearable devices (46). Reyes-Gibby et al. identified the presence of pre-treatment pain as an independent predictor of OCC/OPC 5-year survival (47). According to features importance results, our study highlighted the importance of baseline pre-treatment pain score and vital signs (e.g., weight and heart rate) for contribution in predicting pain intensity, analgesic efficacy, and the total MEDD by GBM and RF models, which is consistent with previous studies (1, 44, 46).

From a clinical decision-making perspective, our study demonstrated that the GBM predicting pain severity at the end of RT provides the highest net benefit at lower to moderate thresholds (0.01–0.31), making it most effective for early identification of high-risk patients who may require preemptive pain management. Clinicians should consider using lower thresholds to guide pain management decisions, ensuring that patients at risk of severe pain receive appropriate interventions before escalation. Additionally, the LR model can help stratify patients based on pain management needs, optimizing decisions regarding intensifying treatment and reducing unnecessary opioid use. By selecting an optimal threshold, clinicians can personalize care, improve patient outcomes, and avoid overtreatment, as higher thresholds may lead to over-prescribing analgesics. The RF model, when predicting analgesic efficacy, performs well at lower thresholds, identifying patients who may benefit from increased pain management while minimizing unnecessary treatments. This approach enhances evidence-based pain management in clinical settings, ensuring effective and individualized treatment.

The clinical importance of early identification of severe pain in HNC cannot be overstated. So far, it is extremely challenging for clinicians to predict pain severity and identify high risk patients depending on their empirical knowledge. Most clinicians prescribe opioids to OCC/OPC patients during therapy according to the pain intensity reported by patients the day of examination, and up to 40% of patients will continue to be dependent on opioids chronically for several months post-therapy (15, 48). Pain control during RT in these cancer populations is still challenging and needs further investigations. This study not only demonstrated the predictive capabilities of ML models but also highlighted their potential clinical applications. These models can aid in risk stratification, allowing for personalized pain management plans based on individual patient characteristics. The use of these ML models in clinical settings could significantly revolutionize pain management strategies for HNC patients undergoing RT, optimizing opioid use and minimizing unnecessary treatments. Our study shows that models like the GBM and RF are particularly valuable in predicting pain severity at the end of RT, offering high net benefits at lower to moderate thresholds. These models can help clinicians identify high-risk patients early, ensuring that those at risk for severe pain receive preemptive pain management before escalation. By utilizing the LR model for MEDD stratification, clinicians can make more informed decisions about the intensity of pain management required, thereby reducing the potential for opioid overprescription. These predictive capabilities also enable clinicians to personalize care, tailoring opioid dosages to individual patient needs and minimizing the risk of chronic opioid dependence post-treatment. With the ability to forecast pain severity and opioid requirements more accurately, ML models offer an opportunity to intervene proactively, improving pain control during RT and potentially reducing the long-term consequences of opioid misuse. The integration of AI and ML in clinical practice can therefore enhance treatment outcomes, optimize pain management protocols, and ultimately improve the QoL for OC/OPC patients, ensuring both more effective and individualized pain relief strategies.

## Limitations

Despite promising findings, this study has limitations. Its retrospective, single-institutional design and reliance on electronic health records introduce biases. To enhance the generalizability of our findings, future studies should focus on validating these ML models using multicentric and prospective cohorts. Additionally, prospective validation would enable real-time assessment of model performance in clinical practice, ensuring that predictions remain accurate and clinically relevant. Patient drop-off due to missing data reduced the final cohort size, necessitating a larger, multicentric cohort for validation. Future work will address external validation of the models on independent datasets, which is crucial to assess model generalizability and robustness. Prospective studies and additional clinical variables may refine predictive performance. Outcome assessment relied on patient-reported pain scores and prescription notes in electronic health records; objective pain assessment methods and data on opioid usage are needed. Acknowledging these limitations is vital for responsible AI/ML implementation in clinical settings.

## Conclusion

This study demonstrates that ML models like GBM, RF, SVM, and LR show promise in risk stratification and predicting acute pain intensity, total MEDD, and analgesic efficacy post-RT. Key predictors include baseline pain intensity and vital sign changes, highlighting the need for early high-risk patient identification for personalized pain management.

## Data Availability

The datasets presented in this study can be found in online repositories. The names of the repository/repositories and accession number(s) can be found below: Data sharing statement: An anonymized version of the data set, including clinical variables, pain scores and opioid dose is available at doi: 10.6084/m9.figshare.25114601. A version of python codes used for data analysis is available at doi: 10.6084/m9.figshare.25713636.
