# Supplementary material for: Machine learning predicting acute pain and opioid dose in radiation treated oropharyngeal cancer patients
Source: Front Pain Res (Lausanne). 2025 Apr 4;6:1567632. doi: 10.3389/fpain.2025.1567632 (PMC12006146; doi:10.3389/fpain.2025.1567632)
Supplement: Supplementary file 1 [file Table1.docx]

**Table 1:** Patients characteristics stratified by acute pain intensity and total MEDD.

| **Variable** | **n** | **SD / %** | **None-Severe pain** | **Severe pain** | **P-value** | **Low MEDD** | **High MEDD** | **P-value** |
| --- | --- | --- | --- | --- | --- | --- | --- | --- |
| **Total** | 900 |  |  |  |  |  |  |  |
| Age (SD) | 60.65 | 9.8 |  |  | 0.0004* |  |  | 0.008* |
| **Sex (%)** |  |  |  |  | 0.27 |  |  | 0.009* |
| Males | 766 | (85%**%)** | 459 (51%) | 307 (34%) |  | 416 (46%) | 350 (39%) |  |
| Females | 134 | (15%**)** | 87 (10%) | 47 (5%) |  | 89 (10%) | 45 (5%) |  |
| **Race (%)** |  |  |  |  | 0.3 |  |  | 0.9 |
| White or Caucasian | 814 | (90%) | 497 (55%) | 317 (35%) |  | 459 (51%) | 355 (39%) |  |
| Black or African American | 25 | (3%) | 10 (1%) | 15 (2%) |  | 15 (2%) | 10 (1%) |  |
| Asian | 16 | (2%) | 12 (1.3%) | 4 (0.7%) |  | 8 (1%) | 8 (1%) |  |
| American Indian or Alaskan Native | 4 | (0.4%) | 2 (0.2%) | 2 (0.2%) |  | 2 (0.2%) | 2 (0.2%) |  |
| Other/unknown | 41 | (4.6%) | 25 (2.7%) | 16 (1.3%) |  | 21 (2.3%) | 20 (2.3%) |  |
| **Smoking (%)** |  |  |  |  | 0.95 |  |  | 0.17 |
| Current smoker | 76 | (8.4%) | 46 (5.1%) | 30 (3.3%) |  | 41 (4.6%) | 35 (3.8%) |  |
| Former smoker | 352 | (39.2%) | 216 (24.1%) | 136 (15.1%) |  | 185 (20.6%) | 167 (18.6%) |  |
| Never smoker | 471 | (52.3%) | 284 (31.5%) | 187 (20.8%) |  | 278 (30.9%) | 193 (21.4%) |  |
| NA | 1 | (0.1) |  |  |  |  |  |  |
| **Alcohol (%)** |  |  |  |  | 0.67 |  |  | 0.175 |
| Yes | 618 | (69.4%) | 371 (41.6%) | 247 (27.7%) |  | 354 (39.8%) | 264 (29.6%) |  |
| No | 273 | (30.6%) | 168 (18.9%) | 105 (11.8%) |  | 143 (16%) | 130 (14.6%) |  |
| NA | 9 | (1%) |  |  |  |  |  |  |
| **Drug abuse (%)** |  |  |  |  | <0.0001* |  |  | 0.007* |
| Yes | 204 | (22.7%) | 93 (10.3%) | 111 (12.4%) |  | 97 (10.7%) | 107 (12%) |  |
| No | 686 | (76.2%) | 445 (49.9%) | 241(27.1%) |  | 400 (44.2%) | 286 (32%) |  |
| NA | 10 | (1.1%) |  |  |  |  |  |  |
| **Clinical-T stage (%)** |  |  |  |  | 0.111 |  |  | 0.319 |
| Tx | 10 | (1%) | 10(1%) | 0(0%) |  | 7(0.7%) | 3(0.3%) |  |
| T0 | 53 | (6%) | 33 (4%) | 20 (2%) |  | 34 (4%) | 19 (2%) |  |
| T1 | 279 | (31**%**) | 170 (19%) | 109 (12%) |  | 164 (18%) | 115 (13%) |  |
| T2 | 297 | (33**%**) | 176 (19.6%) | 121 (13.4%) |  | 166 (18%) | 131 (15%) |  |
| T3 | 147 | (16**%**) | 89 (10%) | 58 (6%) |  | 72 (8%) | 75 (8%) |  |
| T4 | 113 | (13**%**) | 68 (8%) | 45 (5%) |  | 61 (7%) | 52 (6%) |  |
| **Clinical-N stage (%)** |  |  |  |  | 0.63 |  |  | 0.663 |
| NX | 4 | (0.4%) | 2 (0.2%) | 2 (0.2%) |  | 2 (0.2%) | 2 (0.2%) |  |
| N0 | 134 | (15**%**) | 85 (10%) | 49 (5%) |  | 83 (9%) | 51 (6%) |  |
| N1 | 445 | (49.4**%**) | 259 (28.8%) | 189 (20.6%) |  | 246 (27.2%) | 199 (22.2%) |  |
| N2 | 286 | (32**%**) | 181 (20%) | 105 (12%) |  | 158 (18%) | 128 (14%) |  |
| N3 | 31 | (3.2**%**) | 19 (2.1%) | 12 (1.1%) |  | 16 (1.6%) | 15 (1.6%) |  |
| **Primary tumor type (%)** |  |  |  |  | 0.049* |  |  | 0.328 |
| Oral cavity | 100 | (11%) | 70 (7.7%) | 30 (3.3%) |  | 63 (7%) | 37 (4%) |  |
| Oropharynx | 772 | (86%) | 456 (51%) | 35%) |  | 427 (47%) | 345 (39%) |  |
| Unknown primary | 28 | (3%) | 20 (2.2%) | 8 (0.8%) |  | 15 (1.6%) | 13 (1.4%) |  |
| **Chemotherapy (%)** |  |  |  |  | 0.024* |  |  | 0.481 |
| Yes | 641 | (71**%**) | 374 (41.6%) | 267 (29.4%) |  | 355 (39%) | 286 (32%) |  |
| No | 259 | (29**%**) | 172 (19%) | 87 (10%) |  | 150 (17%) | 109 (12% |  |
| Surgery (%) |  |  |  |  | 0.0003* |  |  | 0.002* |
| Yes | 283 | (31%) | 196 (21%) | 87 (10%) |  | 180 (20%) | 103 (11%) |  |
| No | 617 | (69%) | 350 (39%) | 267 (30%) |  | 325 (36%) | 292 (32%) |  |
| **Proton therapy (%)** |  |  |  |  | 0.28 |  |  | 1.3 |
| Yes | 143 | (16%) | 81 (9%) | 62 (7%) |  | 74 (8%) | 69 (8%) |  |
| No | 757 | (84%) | 465 (52%) | 292 (32%) |  | 431 (48%) | 326 (36%) |  |
| Pre-RT pain (mean, SD) | 2.2 | 2.8 |  |  | <0.0001* |  |  | <0.0001* |
| Change in weight (mean%, SD) | -6.6 | 5.8 |  |  | 0.13 |  |  | 0.002* |
| Change in pulse (mean, SD) | 13.8 | 17.3 |  |  | 0.001* |  |  | 0.29 |
| Total MEDD (mean, SD) | 52 | 46 |  |  | <0.0001* |  |  |  |
| Last week Pain score (mean, SD) | 5.3 | 2.7 |  |  |  |  |  | <0.0001* |
| Abbreviations: n: number, SD: Standard Deviation  *Significant difference <0.05 | | |  |  |  |  |  |  |
